# Supplementary figures and images for: Epigenome-based splicing prediction using a recurrent neural network
Source: PLoS Comput Biol. 2020 Jun 25;16(6):e1008006. doi: 10.1371/journal.pcbi.1008006 (PMC7343189; doi:10.1371/journal.pcbi.1008006)

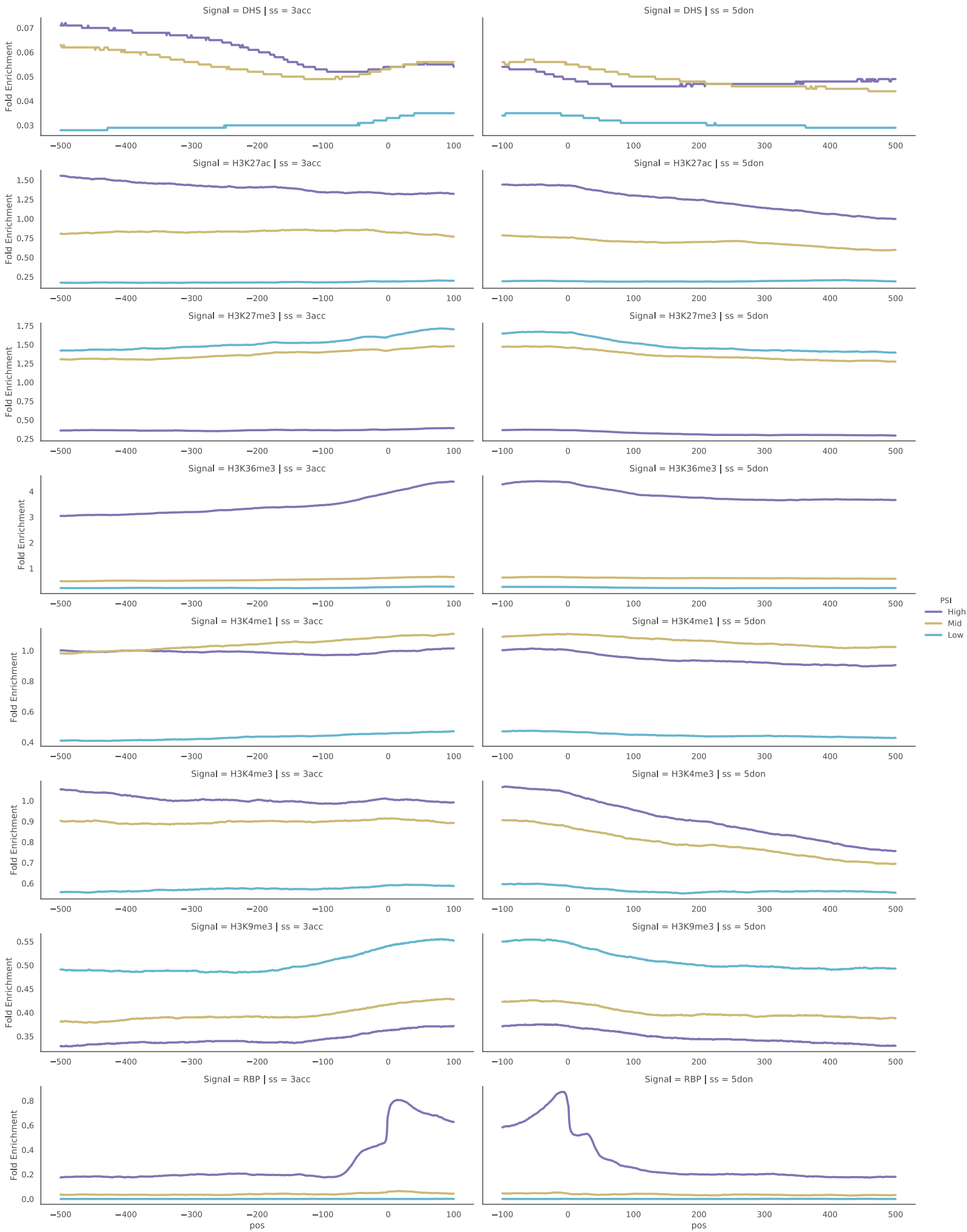

Supplement: S1 Fig — High PSI indicates exon inclusion, mid PSI indicates exons with 40–60% PSI, and low PSI indicates exon skipping. (PDF) [file pcbi.1008006.s005.pdf]

**A**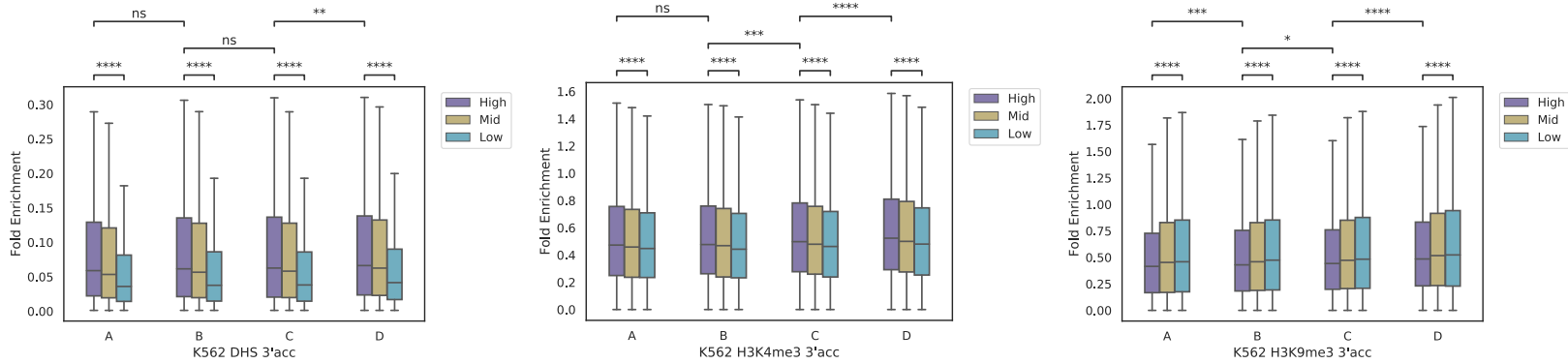**B**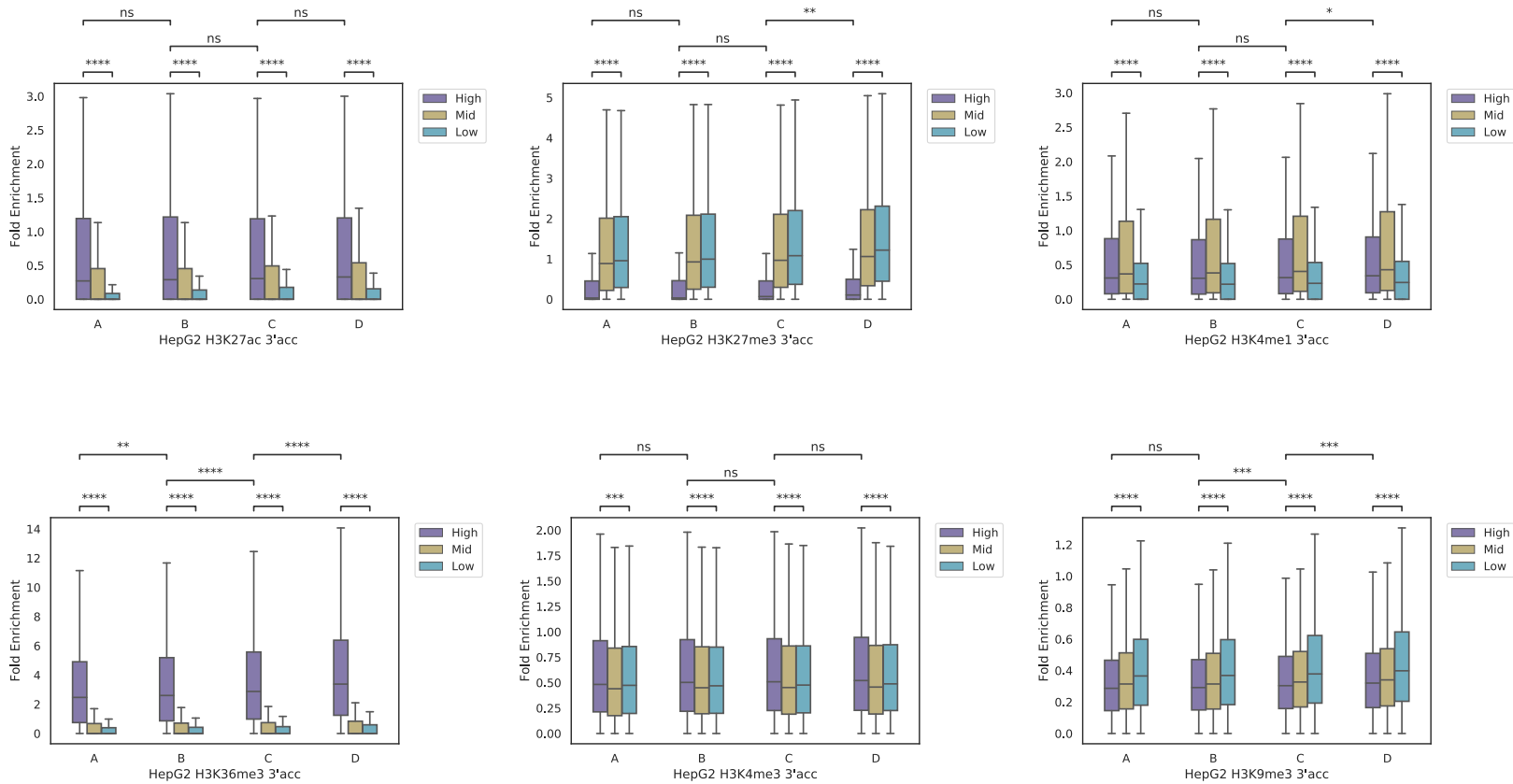**C**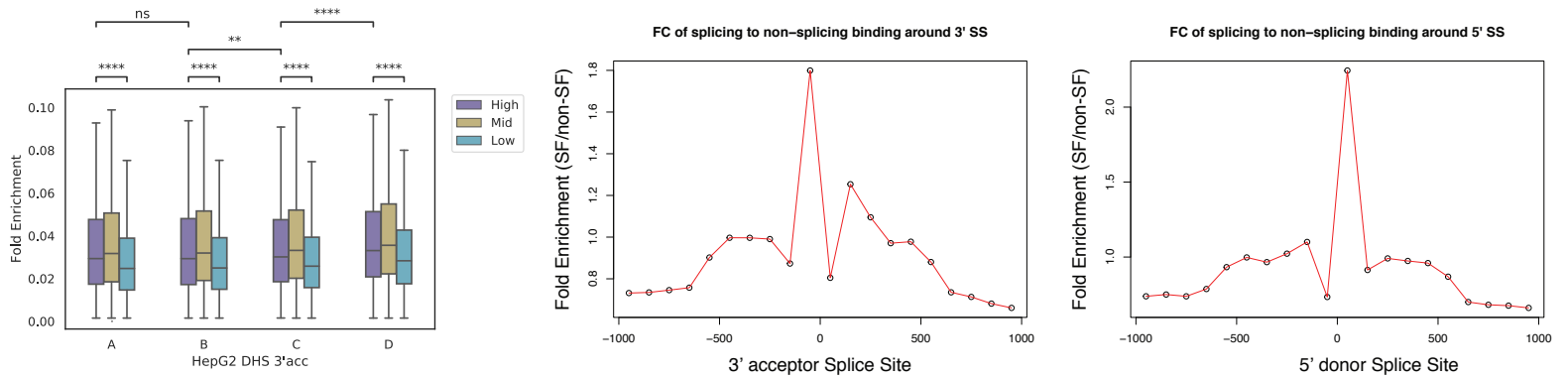

Supplement: S2 Fig — (Shadow figure of the main Fig 2B) Comparison of epigenetic enrichment around different segments of the 3’ acceptor site for (A) K562 and (B) HepG2. High PSI indicates exon inclusion, mid PSI indicates exons with 40–60% PSI, and low PSI indicates exon skipping. Mann-Whitney-Wilcoxon two-sided test, ns: 0.05 < p < = 1; *: 0.01 < p < = 0.05; **: 0.001 < p < = 0.01; ***: 0.0001 < p < = 0.001; ****: p < = 0.0001. (C) Fold enrichment of splicing-related RBPs to non-splicing-related RBPs around the 3’ acceptor splice site and 5’ donor splice site. (PDF) [file pcbi.1008006.s006.pdf]

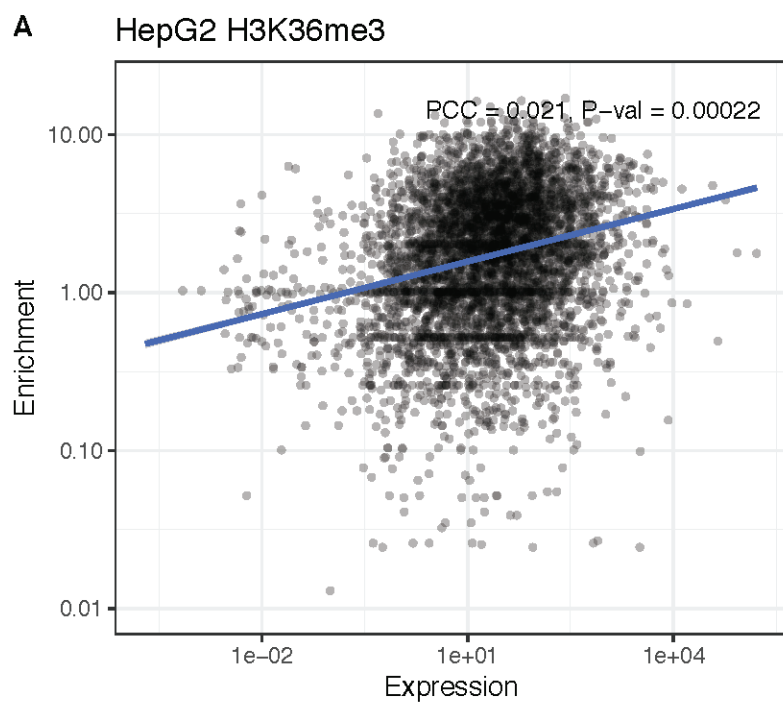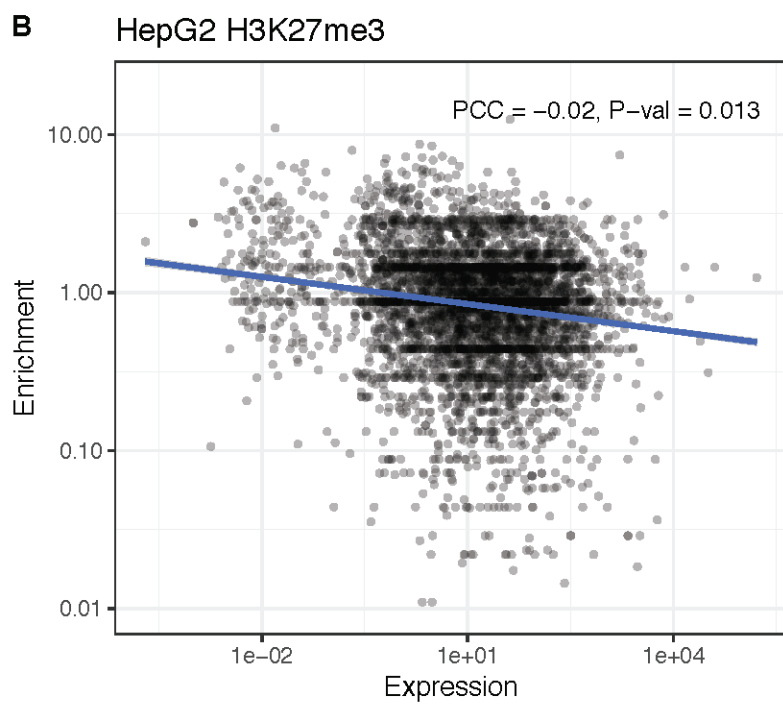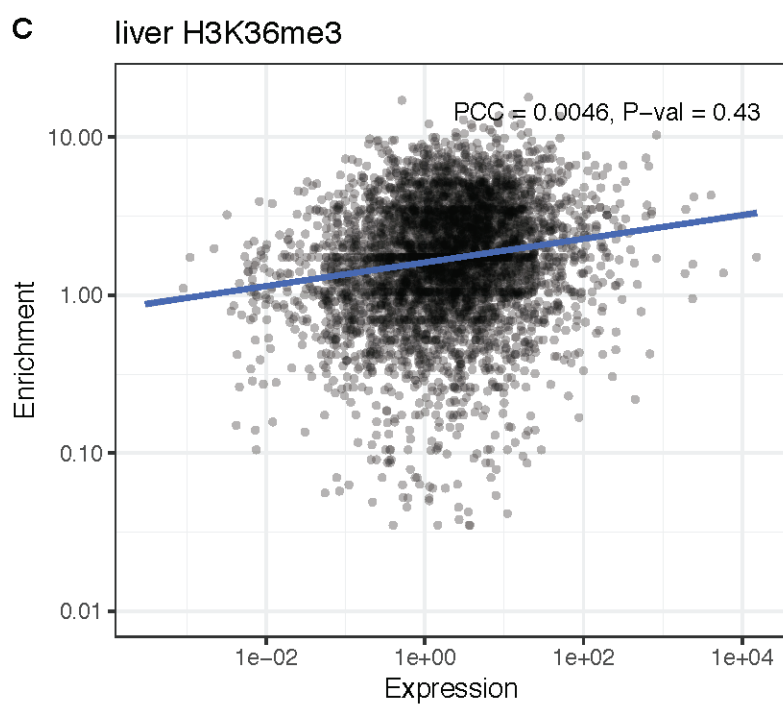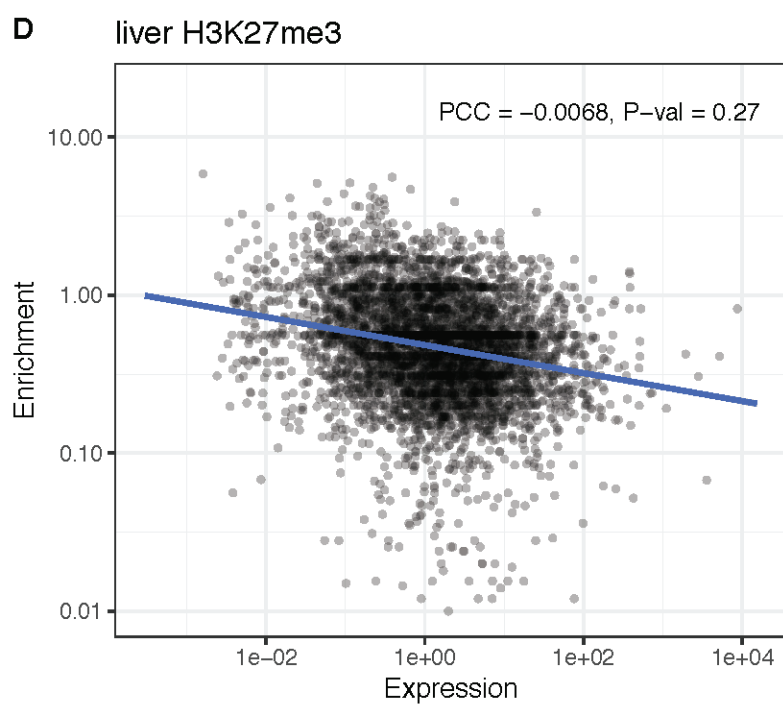

Supplement: S3 Fig — Correlation of exonic expression (FPKM) and histone enrichment of (A) HepG2 H3K36me3, (B) HepG2 H3K27me3, (C) liver H3K36me3, and (D) liver H3K27me3. PCC: Pearson Correlation Coefficient. (PDF) [file pcbi.1008006.s007.pdf]

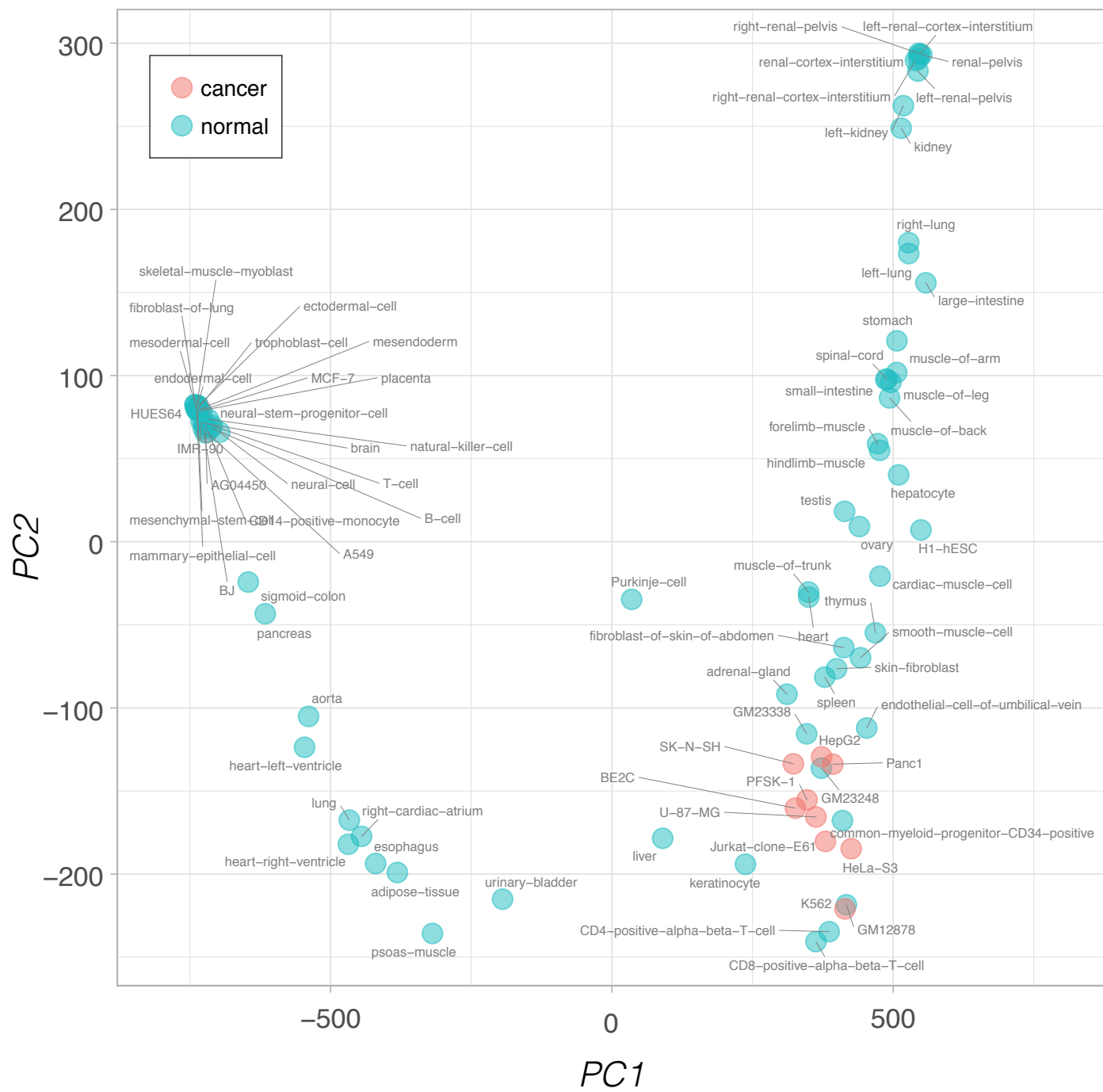

Supplement: S4 Fig — (PDF) [file pcbi.1008006.s008.pdf]

**A**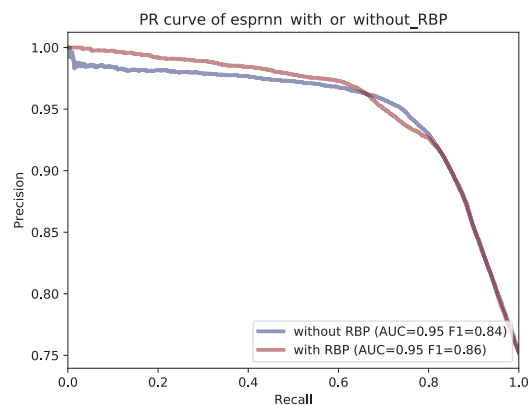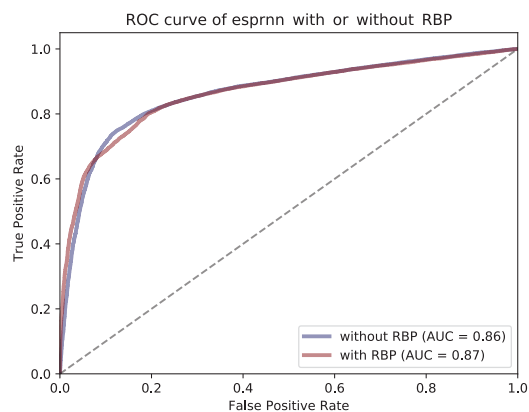**B**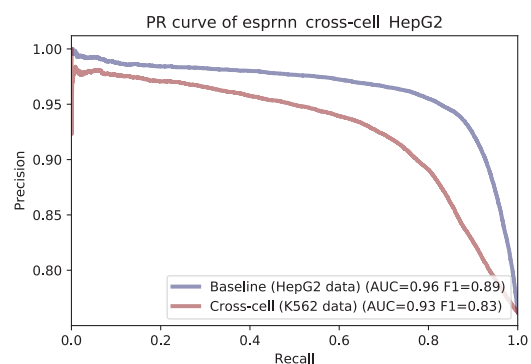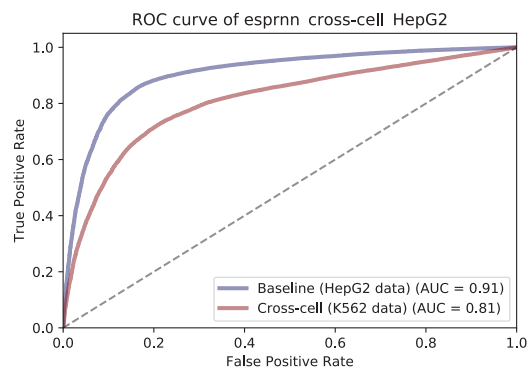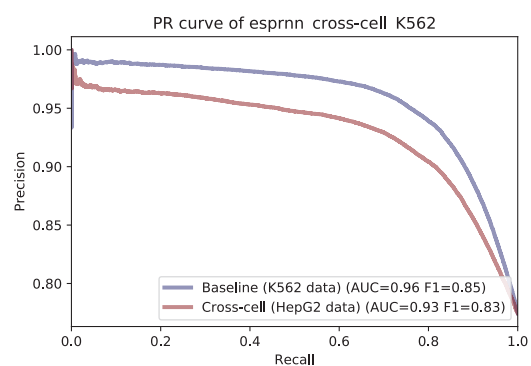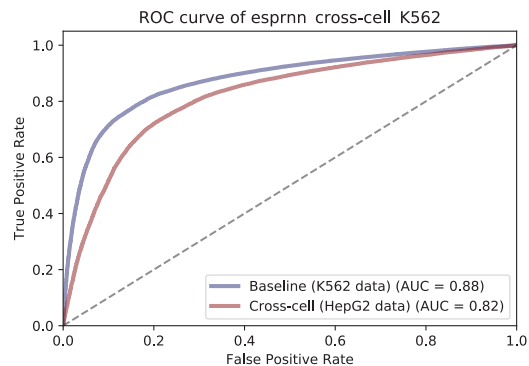

Supplement: S5 Fig — (A) Difference in splicing prediction performance when RBP binding profiles were added as an additional feature of the base model containing chromatin accessibility and histone marks. (B) Cross-cell testing of model. Model was trained on HepG2 data and tested on K562 data, and vice versa. (PDF) [file pcbi.1008006.s009.pdf]

**A**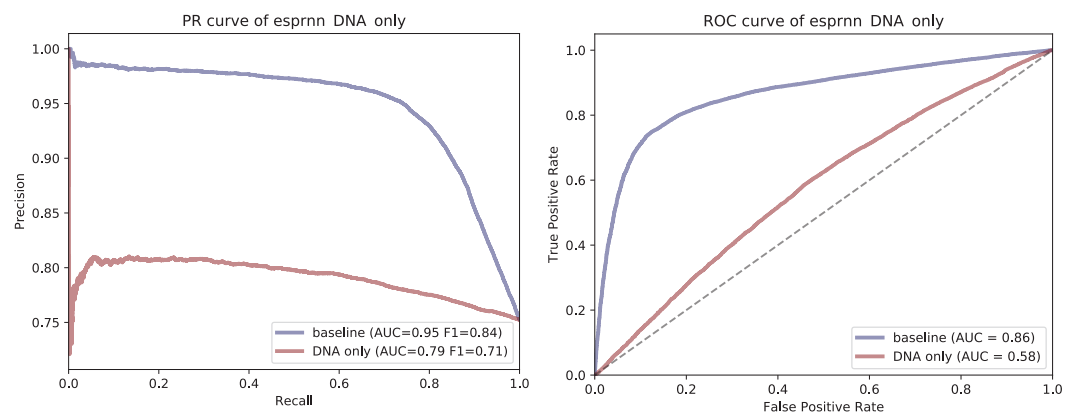**B**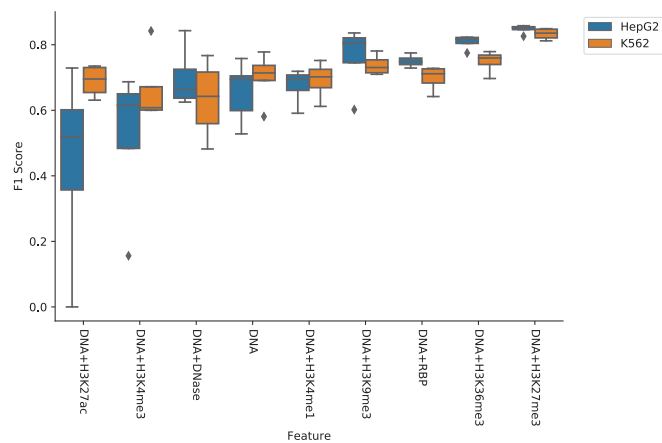**C**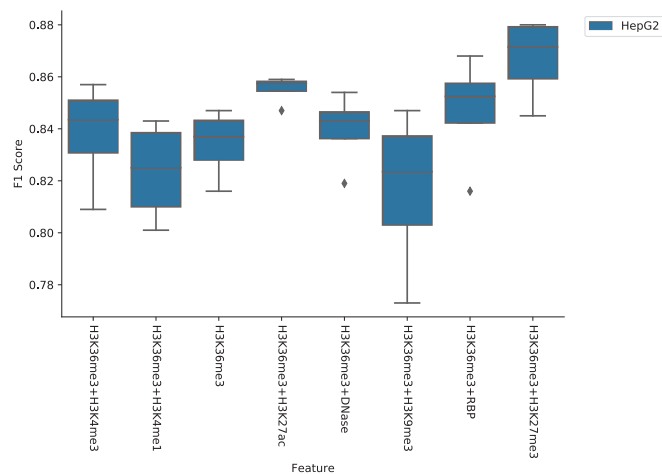**D**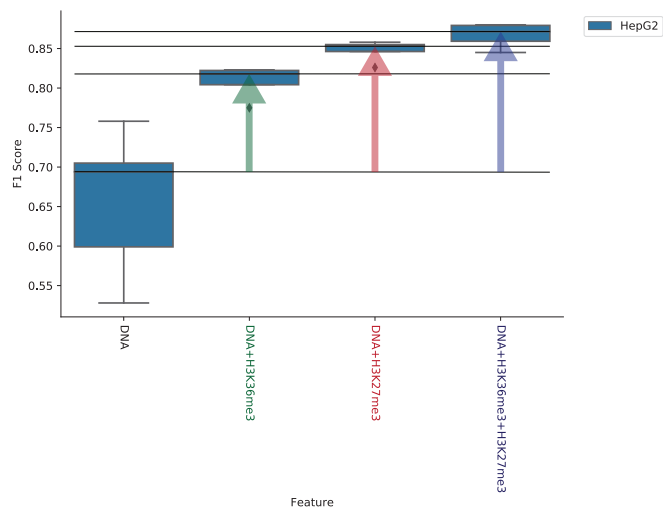

Supplement: S6 Fig — (A) Comparison of the baseline model trained using chromatin accessibility and 6 histone marks to a model using DNA sequence feature only (B) Measure of information gain from additional epigenetic feature based on DNA sequence only model (C) Comparison of splicing prediction performance using a pair of epigenetic features. (D) Performance comparison of models using H3K36me3 or H3K27ac feature individually to a model using both H3K36me3 and H3K27ac features. Performance was measured based on F1 score from 5 trials. (PDF) [file pcbi.1008006.s010.pdf]

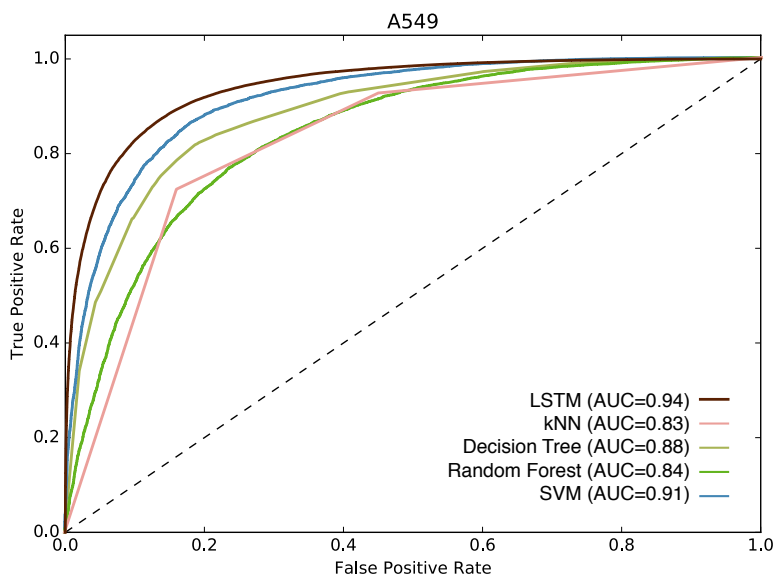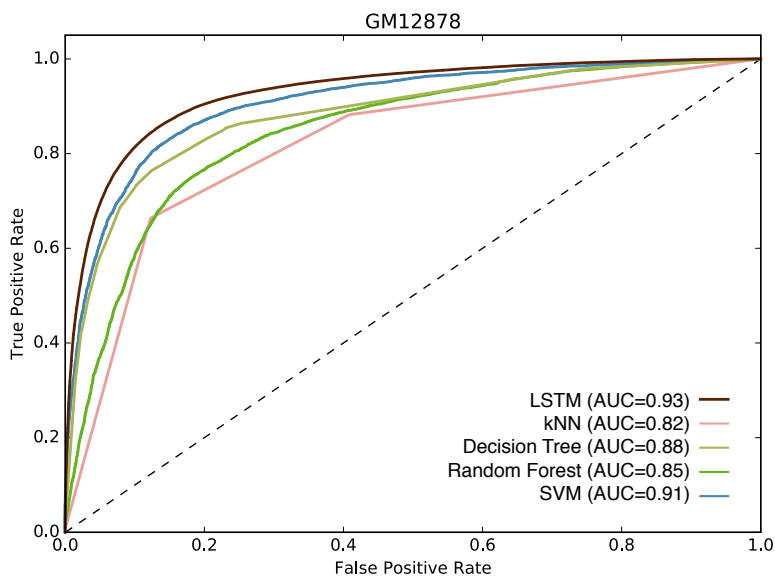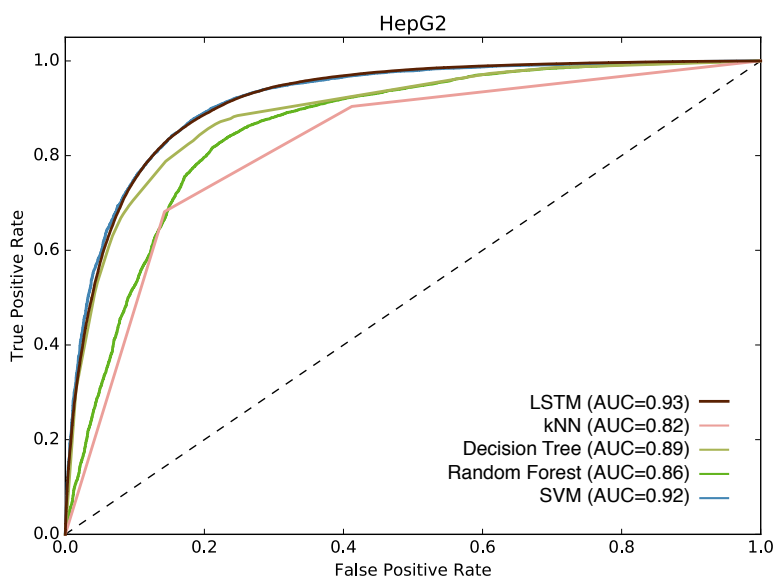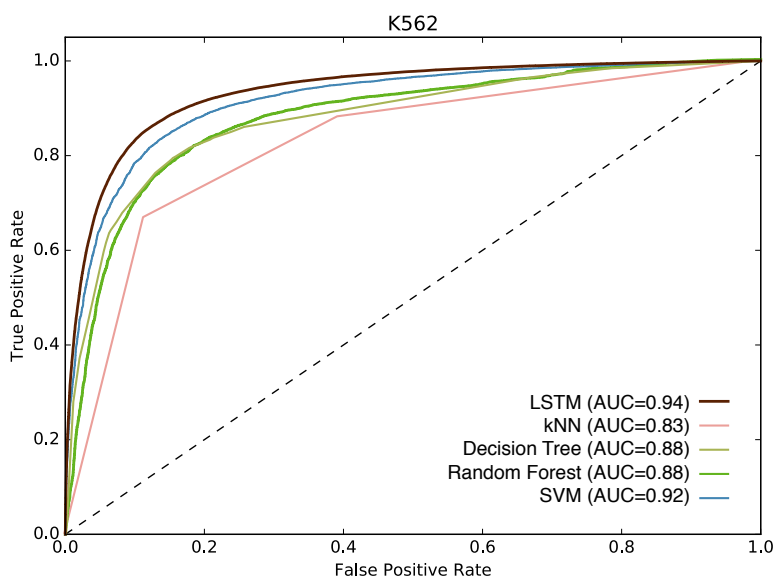

Supplement: S7 Fig — Four different algorithms, k-Nearest neighbor (kNN), decision tree, random forest, and support vector machine (SVM), were compared to the LSTM-based model across four different tissue types (A549, HepG2, GM12878, K562). (PDF) [file pcbi.1008006.s011.pdf]

**A**

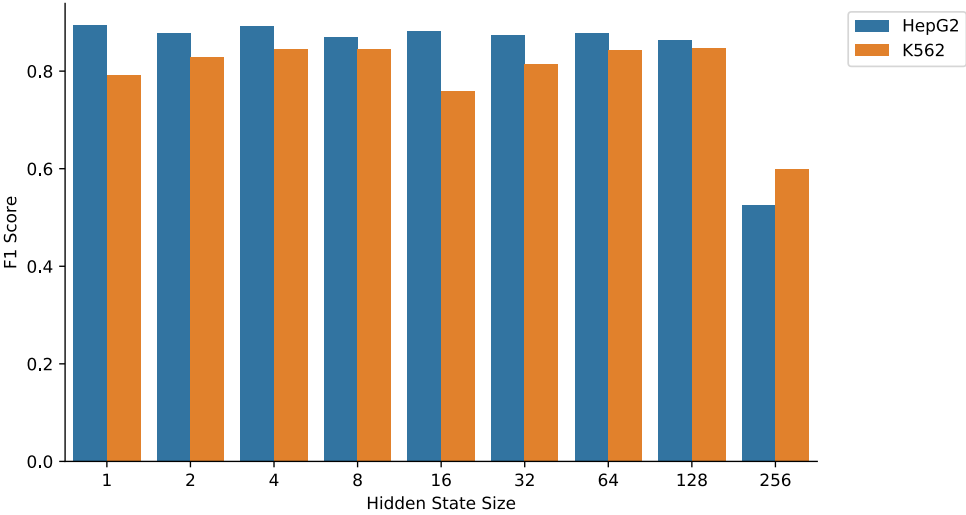

**B**

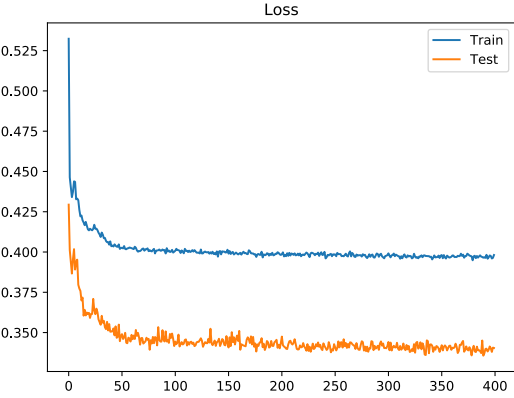

**C**

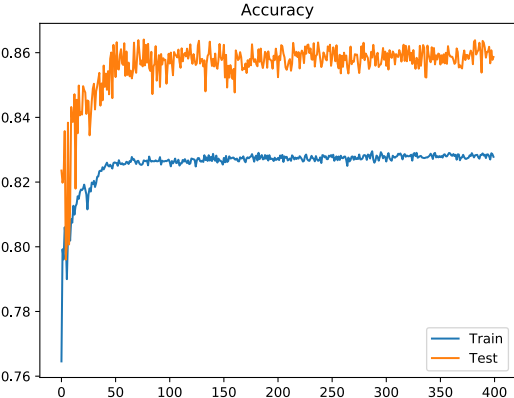

**D**

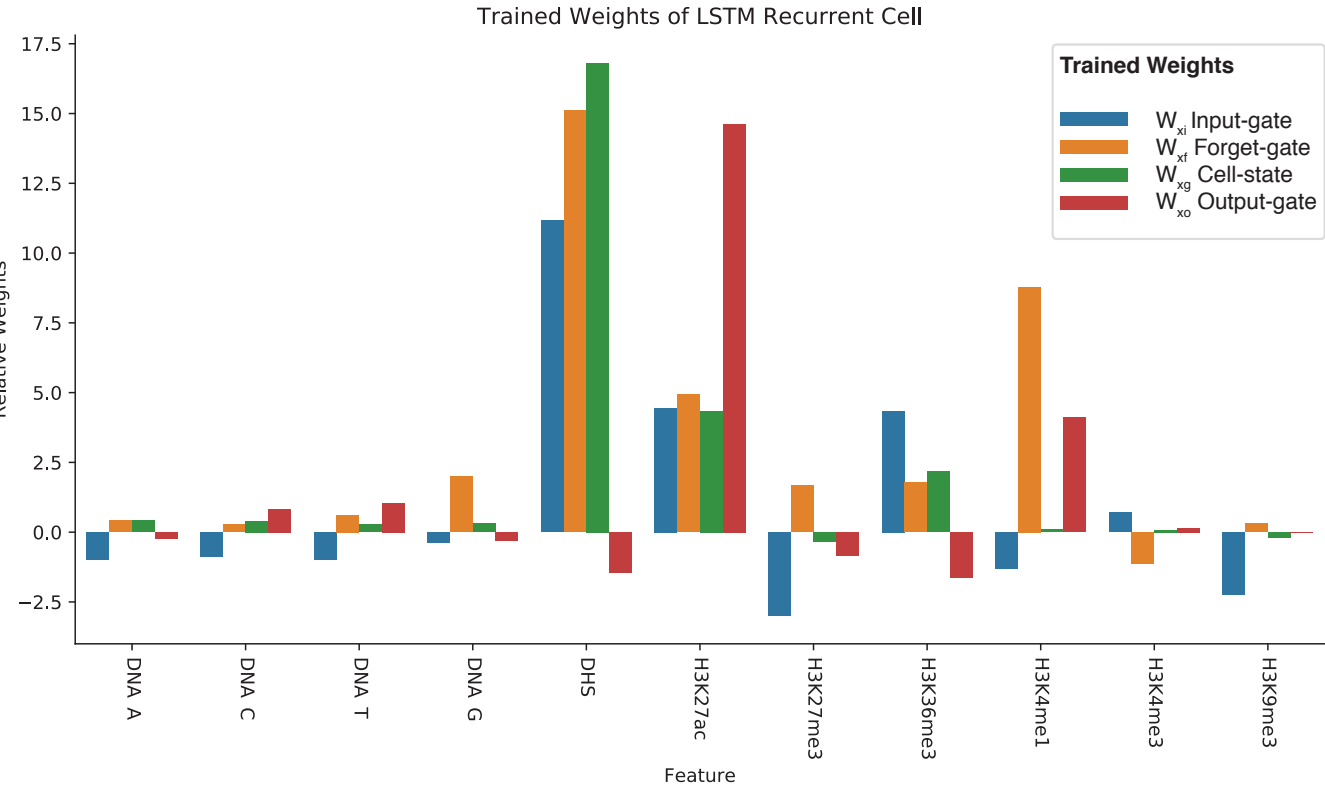

Supplement: S8 Fig — (A) Comparison of splicing prediction performance across different sizes of hidden state. (B) Loss of training an LSTM model with 1 hidden layer for 400 epochs. (C) Accuracy of training an LSTM model with one hidden layer for 400 epochs. (D) Trained weights of LSTM recurrent cells. (PDF) [file pcbi.1008006.s012.pdf]

**A**

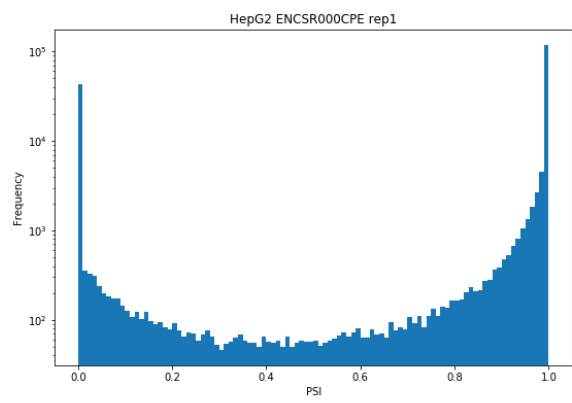

**B**

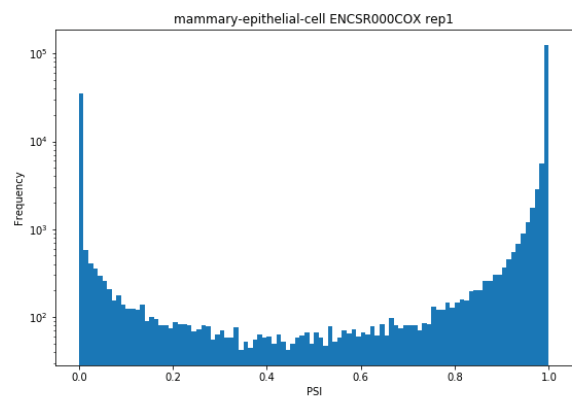

**C**

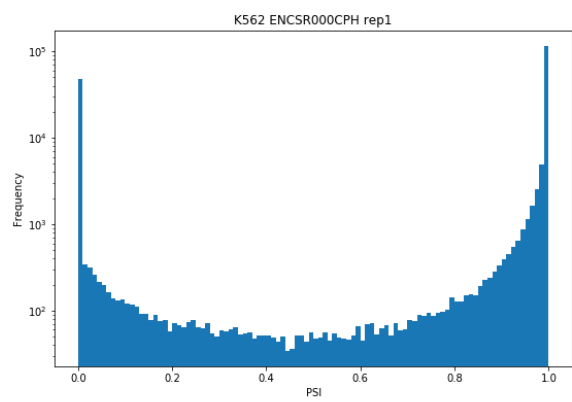

**D**

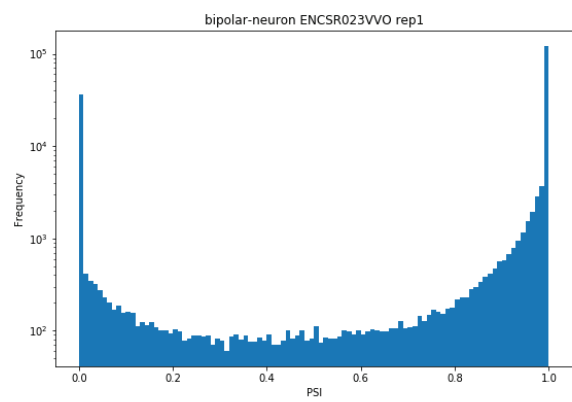

Supplement: S10 Fig — PSI histogram of cassette exons from (A) HepG2 (B) mammary epithelial cell (C) K562, and (D) bipolar neuron. (PDF) [file pcbi.1008006.s014.pdf]
