# Supplementary material for: Epigenome-based splicing prediction using a recurrent neural network
Source: PLoS Comput Biol. 2020 Jun 25;16(6):e1008006. doi: 10.1371/journal.pcbi.1008006 (PMC7343189; doi:10.1371/journal.pcbi.1008006)
Supplement: S9 Fig — (A) precision-recall curve for HepG2 (B) ROC curve for HepG2 (C) precision-recall curve for K562 (D) ROC curve for K562 (PDF) [file pcbi.1008006.s013.pdf]

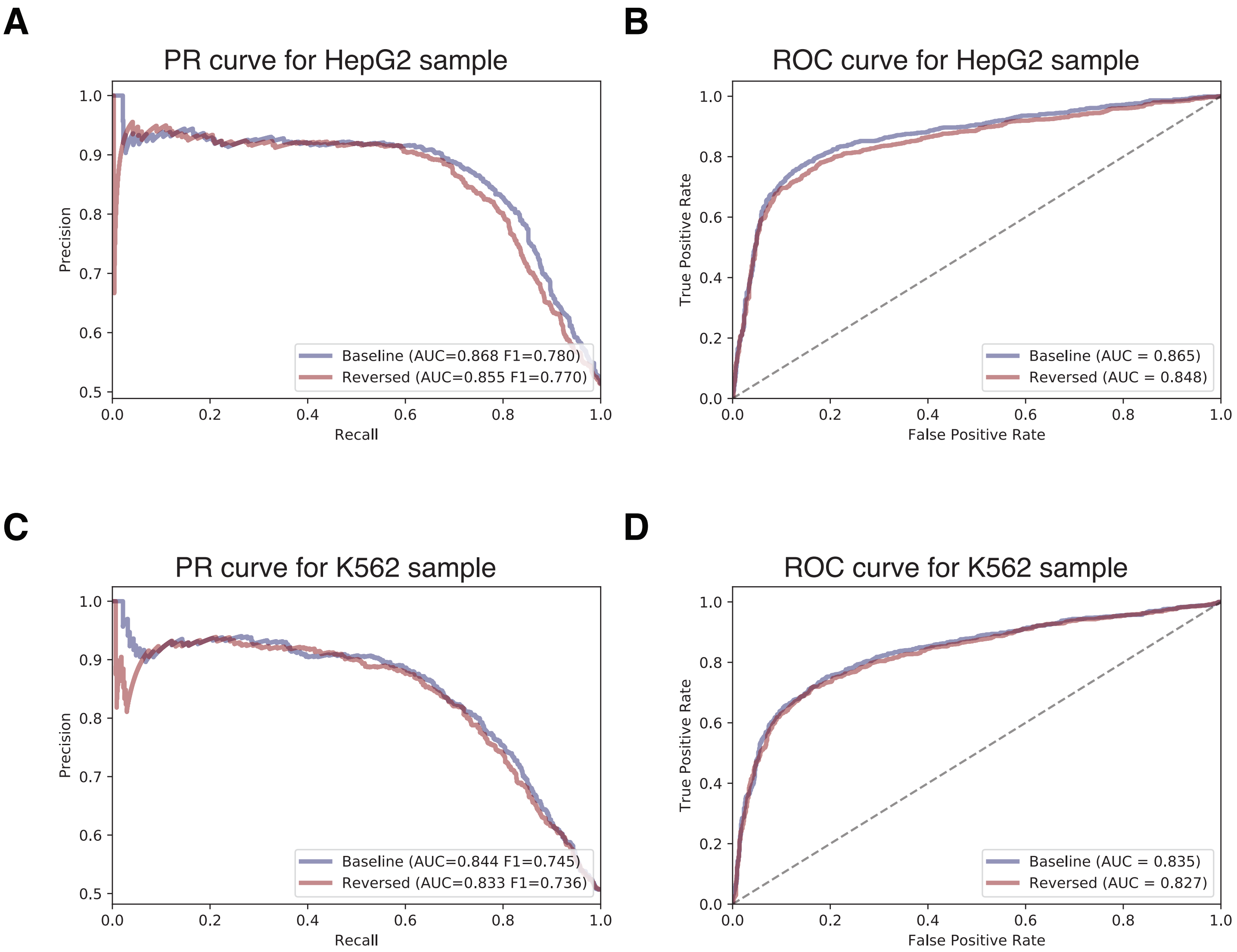
A
PR curve for HepG2 sample
Precision
Recall
Baseline (AUC=0.868 F1=0.780)
Reversed (AUC=0.855 F1=0.770)
B
ROC curve for HepG2 sample
True Positive Rate
False Positive Rate
Baseline (AUC = 0.865)
Reversed (AUC = 0.848)
C
PR curve for K562 sample
Precision
Recall
Baseline (AUC=0.844 F1=0.745)
Reversed (AUC=0.833 F1=0.736)
D
ROC curve for K562 sample
True Positive Rate
False Positive Rate
Baseline (AUC = 0.835)
Reversed (AUC = 0.827)
